# Socioeconomic-related inequalities in oral hygiene behaviors: a cross-sectional analysis of the PERSIAN cohort study

**DOI:** 10.1186/s12903-020-1036-6

**Published:** 2020-02-28

**Authors:** Moslem Soofi, Yahya Pasdar, Behzad Karami Matin, Behrooz Hamzeh, Satar Rezaei, Ali Kazemi Karyani, Mehdi Moradi Nazar, Shahin Soltani, Mohammad Hajizadeh, Yahya Salimi, Alireza Zangeneh, Hossein Poustchi, Maryam Sharafkhah, Ali Akbar Haghdoust, Mahboobeh Shirzad Ahoodashti, Vahid Mohammadkarimi, Javad Aghazadeh-Attari, Fariborz Mansour-Ghanaei, Abbas Yazdanbod, Ebrahim Eftekhar, Zahra Rahimi, Ehsan Bahramali, Alireza Moslem, Ahmad Jamalizadeh, Fatemeh Ezoddini Ardakani, Mehdi Zanganeh, Ali Ahmadi, Alireza Ostadrahimi, Fariba Tohidinezhad, Salar Rahimi Kazerooni, Farid Najafi

**Affiliations:** 1 Social Development and Health Promotion Research Center, Health Institute, Kermanshah University of Medical Sciences, Kermanshah, Iran; 2 Research Center for Environmental Determinants of Health, Health Institute, Kermanshah University of Medical Sciences, Kermanshah, Iran; 3 School of Health Administration, Faculty of Health, Dalhousie University, Halifax, Canada; 4 Liver and Pancreatobiliary Disease Research Center, Digestive Diseases Research Institute, Tehran University of Medical Sciences, Tehran, Iran; 5 Modeling in Health Research Center, Institute for Futures Studies in Health, Kerman University of Medical Sciences, Kerman, Iran; 6 Department of Internal Medicine, Faculty of Medicine, Mazandaran University of Medical Sciences, Sari, Iran; 7 Gastroenterohepatology Research Center, Shiraz University of Medical Sciences, Shiraz, Iran; 8 Social Determinants of Health Research Center, Urmia University of Medical Sciences, Urmia, Iran; 9 Gastrointestinal and Liver Diseases Research Center, Guilan University of Medical Sciences, Rasht, Iran; 10 Digestive Disease Research Center, Ardabil University of Medical Sciences, Ardabil, Iran; 11 Molecular Medicine Research Center, Hormozgan Health Institute, Hormozgan University of Medical Sciences, Bandar Abbas, Iran; 12 Department of Biostatistics and Epidemiology, School of Public Health, Ahvaz Jundishapur University of Medical Sciences, Ahvaz, Iran; 13 Noncommunicable Diseases Research Center, Fasa University of Medical Sciences, Fasa, Iran; 14 Department of Anesthesiology, Sabzevar University of Medical Sciences, Sabzevar, Iran; 15 Vice-Chancellor for Health, Rafsanjan University of Medical Sciences, Rafsanjan, Iran; 16 Department of Oral and Maxillofacial of Radiology, Faculty of Dentistry, Shahid Sadoughi University of Medical Sciences, Yazd, Iran; 17 Health Promotion Research Center, Zahedan University of Medical Sciences, Zahedan, Iran; 18 Modeling in Health Research Center and School of Public Health, Department of Epidemiology and Biostatistics, Shahrekord University of Medical Sciences, Shahrekord, Iran; 19 Nutrition Research Center, Tabriz University of Medical Sciences, Tabriz, Iran; 20 Department of Medical Informatics, Faculty of Medicine, Mashhad University of Medical Sciences, Mashhad, Iran; 21 Colorectal Research Center, Shiraz University of Medical Sciences, Shiraz, Iran

**Keywords:** Socioeconomic inequalities, Oral hygiene behaviors, Concentration index, Decomposition analysis, PERSIAN cohort, Iran

## Abstract

**Background:**

Socioeconomic-related inequality in oral hygiene behaviors in Iran is poorly understood. This study aims to measure and decompose socioeconomic-related inequalities in oral hygiene behaviors among middle-aged and elderly adults in Iran.

**Methods:**

A cross-sectional analysis was performed using data from the Prospective Epidemiological Research Studies in IrAN (PERSIAN), a large national cohort study. A total of 130,016 individuals aged 35 years and above from 17 cohort centers in Iran were included in the study. The normalized concentration index (*C*_*n*_) was used to measure the magnitude of inequality in oral hygiene behaviors, i.e. brushing at least twice and flossing once daily, among middle-aged and elderly Iranian adults included in the cohort centers. Decomposition analysis was performed to quantify the contribution of each determinant to the observed inequality in oral hygiene behaviors.

**Results:**

Totally, 65.5% of middle-aged and elderly adults brushed their teeth twice a day or more, 7.6% flossed at least once a day and 3.48% had both habits. The estimated *C*_*n*_ of the two habits combined, i.e. tooth brushing and dental flossing, for all provinces taken part in the PERSIAN cohort study was 0.399 (95% confidence interval [CI]: 0.383 to 0.417), indicating that the prevalence of the two habits combined is more concentrated among individuals with higher socioeconomic status. Inequality in oral hygiene behaviors was pro-rich in all cohort centers. The decomposition results suggested socioeconomic status as the main factor contributing to the overall inequality, followed by the level of education, and the province of residence.

**Conclusion:**

A low prevalence of oral hygiene behaviors among middle-aged and elderly Iranian adults was observed. There was also a pro-rich inequality in oral hygiene behaviors among middle-aged and elderly adults in all cohort centers. These results suggest an urgent need for targeted policy interventions to increase the prevalence of preventive oral hygiene behaviors among the poor and less-educated middle-aged and elderly adults in Iran.

## Background

Dental problems are major public health concerns worldwide [1] and have negative consequences on the quality of life [2]. Oral and dental problems impose a substantial economic burden on individuals, their families as well as the health system. Treatments of oral diseases are costly, especially for low-income and deprived households [1]. Direct and indirect costs of oral diseases account for approximately 7% of the total health expenditures, implying the importance of oral hygiene behaviors for oral disease prevention [3]. Although oral and dental problems can be avoided by appropriate oral hygiene behaviors and preventive self-care practices, these problems continue to persist in many countries around the world [4, 5].

It has been shown that oral hygiene is a cost-effective and self-performed preventive strategy in improving oral health conditions. Proper oral health behaviors such as tooth brushing, dental flossing and receiving regular dental checkups are effective strategies to prevent tooth decay, and periodontal diseases [5, 6]. According to the American Dental Association (ADA), regular habits of tooth brushing (at least twice a day) and flossing (at least once a day) can effectively prevent oral problems [7]. A systematic review concluded that flossing, in addition to tooth brushing, reduces gingivitis compared to tooth brushing alone [8]. Although compliance with the ADA recommendation of oral hygiene behaviors is highly recommended [7], some studies have shown that a large proportion of individuals brush and floss their teeth less than what is suggested [9, 10]. Poor oral health behaviors, especially poor dental self-cares (e.g. tooth brushing and dental flossing) and non-use of dental service are associated with the dental impairments, and thus reduced oral health-related quality of life [11]. Considerable evidence indicates that the prevalence of dental problems is unequally distributed across socioeconomic groups: individuals with lower socioeconomic status (SES) have a higher burden of dental diseases than their higher SES counterparts [12–15]. Socioeconomic-related inequalities in oral health status have been observed in low-, middle-, and high-income countries [16, 17], so that higher SES individuals clean their teeth more effectively and frequently and use more self-performed preventive strategies [18].

Despite the growing number of studies on socioeconomic-related inequalities in several health indicators, there is a notable paucity of studies measuring socioeconomic inequalities in oral hygiene behaviors. For instance, a study conducted in Iran has indicated a pro-rich inequality in oral hygiene behaviors in Iranian children and adolescents [19]. Although the existing studies in Iran and other countries [9, 15, 20] have assessed the relationship between socioeconomic factors and oral health conditions, these studies did not measure the magnitude of socioeconomic-related inequalities or identify factors that explain such inequalities in dental hygiene behaviors. Specifically, it is very little evidence on daily tooth brushing and dental flossing as specific oral hygiene behaviors among middle-aged and older adults in Iran. Therefore, this study contributes to our understanding of oral hygiene behaviors among middle-aged and older adults in Iran, as most studies in the field have focused on children and adolescents. This study aimed to quantify and decompose socioeconomic inequalities in oral hygiene behaviors (tooth brushing and dental flossing) among 14 out of the 31 provinces of Iran, covering almost all ethnic groups in all geographic areas. We believe that the data from these provinces have the potential to measure socioeconomic-related inequalities in oral hygiene behaviors at the national level.

## Materials and methods

### Data

A cross-sectional analysis of data from the Prospective Epidemiological Research Studies in IrAN (PERSIAN) was done. The PERSIAN cohort was launched in 2014, originally intended to be conducted in 10 geographically-defined regions. It has now stretched to 19 regions of Iran. We obtained the baseline data of 19 cohort centers from the PERSIAN central office in 2018. These regions were selected based on specific characteristics of each region (e.g., population stability, local disease patterns, exposure to certain risk factors, and causes of death). The PERSIAN Cohort aims to identify the risk factors underlie the most common non-communicable diseases in Iran. Further details about the design and the sampling method of the Cohort can be found elsewhere [21, 22]. The initial sample consisted of 131,813 individuals, of which 1376 individuals were excluded due to incomplete data on dental flossing, tooth brushing, age, etc. In this study, we obtained data from 19 cohort centers located in 14 different provinces in Iran. For the purpose of this study, the regional cohort centers located in the same province considered as the province in which they were located (Appendix). In addition, data on Kohgiluyeh and Boyer Ahmad province were excluded from the analysis. Because at the time of this study, the recruitment phase of its cohort center was ongoing and the sample size was insufficient to include into the model. Finally, we used data from 18 cohort center located in 14 provinces of Iran. Finally we used the data of 130,016 individuals aged 35 and older in the analysis.

### Variables

The outcome of interest in the study, good oral hygiene behaviors, is a binary variable representing whether or not an individual had good oral health behaviors. The outcome variable was defined based on these questions: “How many times per day do you brush your teeth? once daily, twice daily, three times a day, four times and more a day and never”, “Do you use dental floss? yes/no” and “How many times per week do you floss?”. Participants who reported 7 times and more per week, considered as those who flossed at least once a day. Therefore, in this study good oral hygiene behaviors were defined for individuals who did brush their teeth with toothpaste at least twice and flossing once daily. The following variables used as determinants of oral hygiene behaviors in the decomposition analysis: sex (male/female), age (35–44, 45–54, 55–64 and 65^+^: Since the age group of 75–85 years was less than 1 % of the sample, we merged this age group with the age group of 65–75 years and considered them as the age group of 65 and older), marital status (single, married, divorced/widowed), level of education (illiterate, primary, intermediate, secondary, higher), the SES of individuals and province of residence (14 geographically-defined provinces of Iran). The SES of individuals was measured using a constructed household wealth index. The participants had been asked about whether they possessed certain durable assets, including a laptop, dishwashing machine, freezer, three-dimensional TV, vacuum cleaner, car, motorcycle, personal computer/laptop, smartphone, the number of rooms per capita, and the type of homeownership, they also had been asked about their infrastructure facilities (access to internet, access to piped drinking water). Based on these information, we constructed the wealth index using principal component analysis (PCA) technique. Any variables with a frequency of less than 5% or more than 95% were not included in the PCA model. The PCA generates the weight for each selected asset and then estimates a continuous index based on the sum of all weights of the variables included in the PCA for each individual. The index was categorized into wealth quintiles, where the 1st quintile indicates the poorest SES group and the 5th quintile indicates the richest SES group [23–26].

### Statistical analysis

The concentration index (C) was used to quantify the magnitude of socioeconomic inequalities in oral hygiene behaviors. The C is defined with respect to the concentration curve, which plots the cumulative percentage of good oral hygiene behaviors on the horizontal axis and cumulative percentage of individuals ranked in ascending order of SES in the vertical axis. Twice the area between the line of equality (the diagonal) and the concentration curve is defined as the C. The C ranges between − 1 and + 1. When the C is positive good oral hygiene behaviors are more concentrated among high-SES individuals, and there exists pro-rich inequality. When the C is negative, good oral hygiene behaviors are more concentrated among low-SES individuals, and there exists pro-poor inequality the zero value of C indicates that oral hygiene behaviors is equally distributed among the SES groups. The C can be computed using the following formula [27]:

1 $$ C=\frac{2\ast \mathit{\operatorname{cov}}\left({y}_i{r}_i\right)\kern0.5em }{\mu }, $$

Where *μ* is the mean or the proportion of the good oral hygiene behaviors and *y*_*i*_ and *r*_*i*_ represent the good oral hygiene behaviors and fractional rank in the socioeconomic distribution for the *i* th individual, respectively. As the health outcome variable in this study is binary, the maximum and minimum values of the C are not between − 1 and + 1. To address this issue, as per Wagstaff’s suggestion, we normalized the C by multiplying the estimated C by $\frac{1}{1-\mu }$ .

We decomposed the C to quantify the contribution of each determinant to socioeconomic inequalities in oral hygiene behaviors among middle aged and elderly adults in Iran. According to Wagstaff and colleagues [28] for any linear additive regression model linking our health outcome variable, *y* to a set of k determinants, *x*_*k*_ [28]:

2 $$ y=\alpha +{\sum}_k{\beta}_k\ {x}_k+\varepsilon, $$

The C for oral hygiene behaviors, can be decomposed as follows:

3 $$ C={\sum}_k\left(\frac{\beta_k{\overline{x}}_k}{\mu}\right){C}_k+G{C}_{\varepsilon }/\mu . $$

Where ${\overline{x}}_k$ represents the mean of determinant *k*, *x*_*k*_. The *C*_*k*_ is the C for *x*_*k*_ and $\frac{\beta_k{\overline{x}}_k}{\mu }$ is the elasticity of good oral hygiene behaviors with respect to determinant *k*. The elasticity of each determinant demonstrates the sensitivity of good oral hygiene behaviors to changes in the determinant. A positive elasticity means that individuals with this characteristic are more likely to have good oral hygiene behaviors. The *GC*_*ε*_ indicates the generalized C for the error term. The first part in Eq. 3, ${\sum}_k\left(\frac{\beta_k{\overline{x}}_k}{\mu}\right){C}_k$), is the explained component and indicates the contribution of explanatory variables to the overall socioeconomic inequality in good oral hygiene behaviors. The second part of the Equation, *GCε*/*μ*, is an unexplained (residual) component and shows the portion of the C for good oral hygiene behaviors that cannot be explained by the systematic variations in the determinants across SES groups.

The decomposition of the normalized concentration index, *C*_*n*_, can be written as follows [28]:

4 $$ {C}_n=\frac{C}{1-\mu }=\frac{\sum_k\left(\frac{\beta_k{\overline{x}}_k}{\mu}\right){C}_k}{1-\mu }+\frac{G{C}_{\varepsilon }/\mu }{1-\mu } $$

As our outcome variable was a dichotomous variable, we used marginal effects obtained from the logit model in the decomposition analysis to estimate the contributions of the explanatory variables to the *C*_*n*_. All analyses were conducted using STATA software version 14.

## Results

### Descriptive results

A total of 130,016 adults aged 35 years and older with a mean age of 49.37 (standard deviation [SD] = 9.2) years were included in the study, of which 72,071 (55. 4%) were female. Participants belonged to the age group of 35–44 years old account for 35.6% of the entire sample and the majority of the sample was married (90.9%). Also, the illiterate participants accounted for 35.2% of the whole sample (Table 1).

**Table 1:** Prevalence of oral hygiene behaviors in terms of determinant variables among PERSIAN Cohort participants aged 35 and above in 2018

Variables	n (%)	Brushing ≥ twice/day		Flossing ≥ once/day		Both habits/day	
		n	%	n	%	n	%
Sex							
Male	57,945 (44.5)	44,174	61.2	3702	6.4	1753	3.0
Female	72,071 (55.4)	40,999	70.7	6243	8.6	2768	3.8
Age group							
35–44	46,290 (35.6)	26,721	57.7	5104	11.0	2251	4.8
45–54	43,677 (35.5)	28,298	64.7	3412	7.8	1586	3.6
55–65	31,641 (24.3)	23,346	73.7	1253	3.9	595	1.8
65+	8405 (6.4)	6807	80.9	176	2.1	89	1.0
Marital status							
Single	2941(2.2)	1604	54.5	314	10.7	151	5.1
Married	118,206 (90.9)	77,262	65.3	9182	7.7	4128	3.4
Divorced or widowed	8869 (6.8)	6308	71.1	449	5.0	242	2.7
Level of education							
Illiterate	445,843 (35.2)	35,196	76.7	896	1.9	486	1.0
Primary	32,292 (24.8)	21,580	66.8	1580	4.9	745	2.3
Intermediate	17,230 (13.2)	10,670	61.9	1414	8.2	672	3.9
Secondary	18,556 (14.2)	10,149	54.6	2526	13.6	1093	5.8
Higher	16,095 (12.3)	7578	47.8	3558	22.1	1525	9.4
Socioeconomic Status Quintiles							
1st Quintile (Poorest)	26,042 (20)	19,888	76.3	356	1.3	207	0.7
2nd Quintile	26,029 (20)	18,351	70.5	842	3.2	417	1.6
3rd Quintile	25,950 (19)	17,406	67	1342	5.7	647	2.4
4th Quintile	26,092 (20)	16,081	61.6	2266	8.7	985	3.8
5th Quintile ((Wealthiest)	25,839 (19)	13,407	51.8	5139	19.79	2265	8.7
Province of Residence							
Kermanshah	10,068 (7.7)	6289	62.4	599	5.9	265	2.6
Guilan	10,511 (8)	5309	50.5	625	5.9	277	2.6
Fars	22,994 (17.6)	16,530	71.8	775	3.2	399	1.7
East Azerbaijan	14,978 (11.5)	10,040	67	1079	7.2	449	3.0
Mazandaran	10,253 (7.8)	6372	62.1	1263	12.3	555	5.4
Sistan and Balouchestan	8215 (6.3)	5571	67.8	647	7.8	353	4.3
Yazd	9462 (7.2)	6625	70	992	10.4	481	5.0
Kerman	9914 (7.6)	6408	64.6	771	7.8	299	3.0
Khouzestan	9039 (6.9)	6850	75.7	74	0.8	47	0.5
Chaharmahal and Bakhtiari	6664 (5.1)	3872	58.1	1319	19.4	553	8.3
Hormozgan	3339 (2.5)	1948	58.3	25	0.7	13	0.4
West Azerbaijan	3457 (2.6)	2628	76	74	2.4	47	1.3
Ardabil	8192 (6.3)	5305	64.7	915	11.1	393	4.8
Razavi Khorasan	2930 (2.2)	1426	48.6	807	27.5	390	13.3
Total	130,016	85,173	65.5	9945	7.65	4521	3.48

**Fig. 1:**
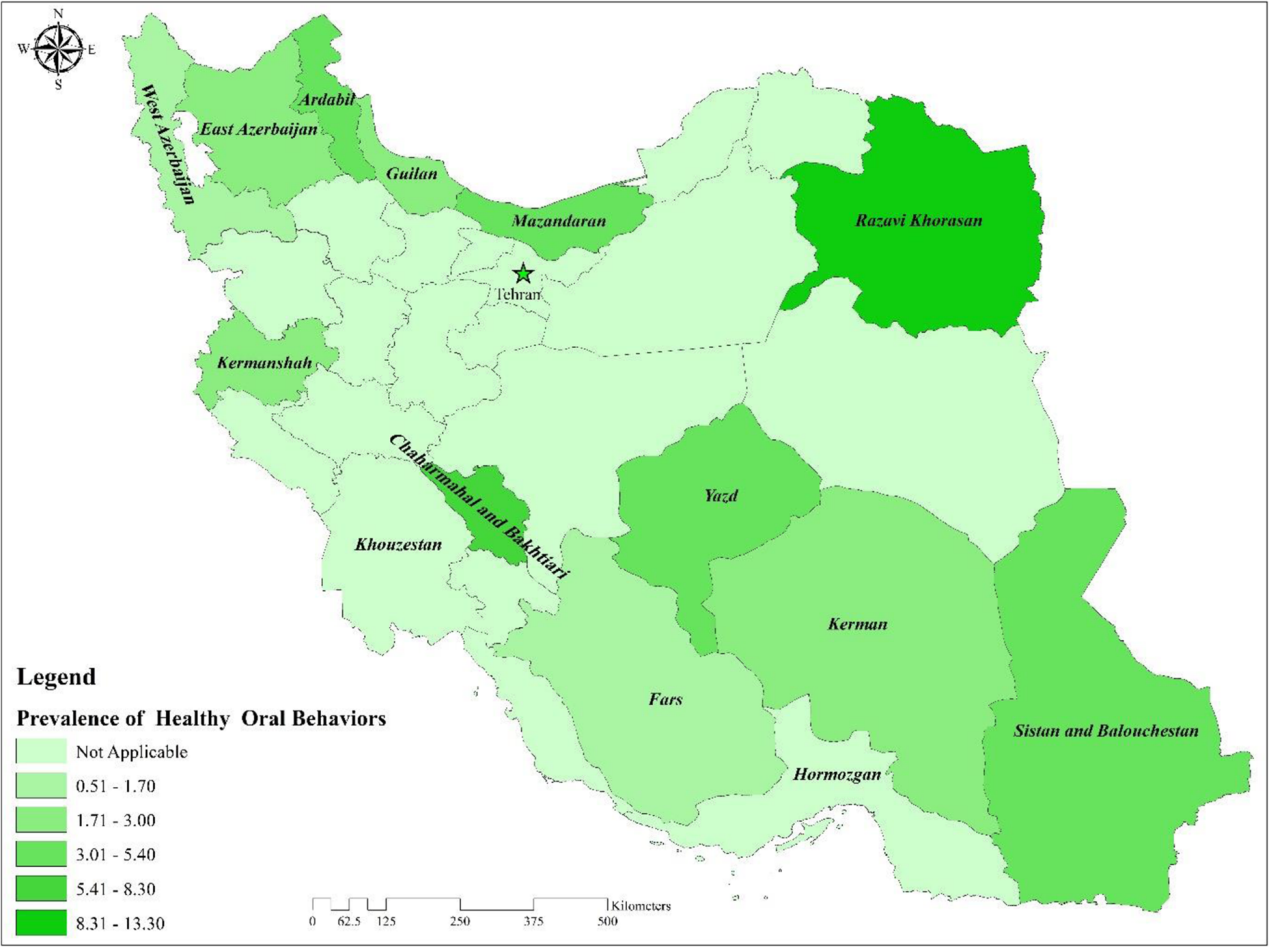
Proportion of oral hygiene behaviors (i.e. twice-daily brushing and once-daily flossing) across cohort centers located in 14 different provinces in Iran, (Source: the findings of the present study)

Totally, 65.5% of adults brushed their teeth twice a day or more, 15.9% reported that they used dental floss, 7.6% flossed at least once a day and 3.4% had both habits. The individuals aged 35–44 years old, married, those with a higher level of education and well-off individuals had a higher prevalence of the two habits combined (i.e. brushing and flossing). The results demonstrated that the cohort of Razavi Khorasan had the highest proportion of the two habits combined (13.3%), followed by the cohort of Chaharmahal and Bakhtiari (8.3%) and Mazandaran (5.4%) provinces. The lowest prevalence was observed in cohort of Khouzestan province (0.5%) (Table and Fig. 1).

### Socioeconomic-related inequalities in oral hygiene behaviors

The estimated *C*_*n*_ was 0.399 (95% confidence interval [CI]: 0.383–0.417) for the entire population, 0.374 (95% CI: 0.347–0.401) for men and 0.427 (95% CI: 0.405–0.449) for women. Positive values of the *C*_*n*_ indicated that the prevalence of good oral hygiene behaviors is more concentrated among high-SES individuals (Table 2).

**Table 2:** Normalized concentration indices for oral hygiene behaviors (brushing at least twice and flossing once daily) among PERSIAN Cohort participants in Iran, 2018

Sample	The *C*_*n*_	Std. error	95% Confidence interval	*P*-value
Total	0.399	0.008	0.383–0.417	0.000
Males	0.374	0.013	0.347–0.401	0.000
Females	0.427	0.011	0.405–0.449	0.000

*C*_*n*_ Normalized concentration index

**Fig. 2:**
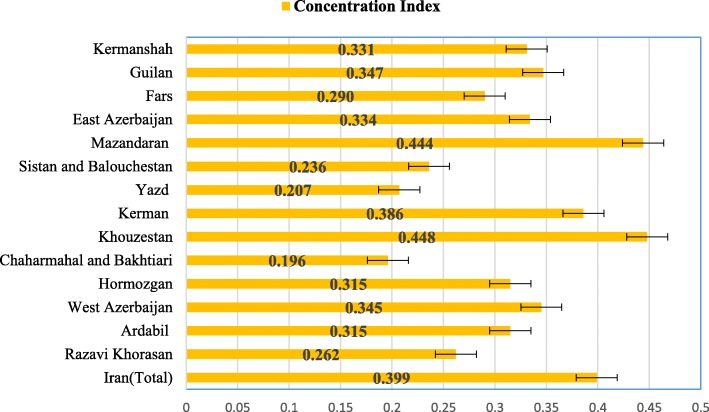
Concentration indices with their 95% confidence intervals for the two oral hygiene behaviors combined across cohort centers located in 14 different provinces in Iran, (Source: the findings of the present study)

The estimated positive value of the *C*_*n*_ for the two habits combined (i.e. brushing and flossing) varied significantly across 17 cohort centers in Iran, suggesting variations in the pro-rich distribution of good oral hygiene behaviors among adult populations living in different provinces/regions. The lowest socioeconomic inequality in oral hygiene behaviors was found in cohort of Chaharmahal and Bakhtiari (*C*_*n*_ =0.196) and the highest socioeconomic inequality was observed in the cohort of Khouzestan (*C*_*n*_ =0.393) followed by cohorts of Mazandaran (*C*_*n*_ =0.408), and Quilan (*C*_*n*_ =0.347) (Fig. 2).

### Determinants of socioeconomic-related inequalities in oral hygiene behaviors

According to the decomposition analysis of the *C*_*n*_, belonging to the age groups of 55–65 and 65+ years, and having higher levels of education attainment positively contributed to the *C*_*n*_, indicating that the socioeconomic distribution of these factors and the association between these factors and oral hygiene behaviors (i.e. brushing at least twice daily and flossing once daily) resulted in the concentration of oral hygiene behaviors among higher SES individuals. Being female contributed negatively to the *C*_*n*_ value, suggesting that the factor concentrated among the lower SES individuals (see the absolute and percentage contribution of these factors reported in Table 3). The main contributor to the pro-rich inequality in good oral hygiene behaviors was SES (69.7%), followed by education level (55.4%), province of residence (11%) and age groups (4.2%) (Table 3).

**Table 3:** Results of the decomposition analysis of socioeconomic inequality in oral hygiene behaviors (brushing at least twice and flossing once daily) among PERSIAN Cohort participants in Iran, 2018

	Marginal Effects	Elasticity	The *C*_*x*_	Contribution		
				Absolute	%	Summed %
Sex						−3.9
Male	Ref.					
Female	0.016	0.257	−0.061	−0.015	−3.9	
Age Group						4.2
35–44	Ref.					
45–54	−0.001	−0.016	0.051	−0.000	− 0.2	
55–65	− 0.013	− 0.091	− 0.098	0.009	2.2	
65+	− 0.016	−0.031	− 0.028	0.009	2.2	
Marital status						−1.9
Single	Ref.					
Married	−0.005	−0.153	0.032	−0.004	−1.2	
Divorced or widowed	0.004	0.008	−0.354	−0.003	− 0.7	
Education level						55.4
Illiterate						
Primary	0.016	0.119	−0.065	−0.007	−1.9	
Intermediate	0.038	0.145	0.110	0.016	4.0	
Secondary	0.052	0.214	0.320	0.068	17.2	
Higher	0.069	0.247	0.583	0.144	36.1	
Socioeconomic Status Quintiles						69.7
Q1(Poorest)	Ref.					
Q2	0.020	0.115	−0.413	−0.047	−11.9	
Q3	0.028	0.162	0.001	0.000	0.0	
Q4	0.036	0.211	0.416	0.087	21.9	
Q5(Wealthiest)	0.050	0.287	0.830	0.238	59.6	
Province of Residence						11.0
Kermanshah	Ref.					
Guilan	−0.001	−0.004	−0.178	0.000	0.1	
Fars	−0.004	− 0.022	−0.377	0.008	2.0	
East Azerbaijan	−0.000	−0.000	0.016	0.000	0.0	
Mazandaran	0.008	0.018	0.133	0.002	0.6	
Sistan and Balouchestan	0.011	0.020	−0.011	−0.000	− 0.0	
Yazd	0.010	0.021	0.169	0.003	0.9	
Kerman	−0.010	−0.022	0.301	−0.006	−1.6	
Khouzestan	−0.027	−0.054	− 0.129	0.007	1.7	
Chaharmahal and Bakhtiari	0.021	0.031	0.442	0.013	3.9	
Hormozgan	−0.030	−0.022	−0.066	0.001	0.3	
West Azerbaijan	−0.014	−0.010	− 0.093	0.001	0.2	
Ardabil	0.007	0.014	0.262	0.003	0.9	
Razavi Khorasan	0.025	0.016	0.575	0.009	2.3	
Total explained				0.538		134.5
Residual				−0.138		--34.5
The *C*_*n*_				0.399		100

## Discussion

In this study, we quantified and decomposed socioeconomic inequalities in oral hygiene behaviors among cohort centers located in 14 different provinces of Iran. There is a current paucity of empirical research focusing specifically on measuring and decomposing socioeconomic inequalities in dental self-care behaviors in the middle-aged and elderly adults at the national level in Iran. Evidence has shown the role of various factors, including dietary habits (e.g. Sugar intake), use of fluoride, regular dental visit and dental self-care practice on oral health status [4, 29]. Among these factors, this study has focused on oral hygiene behaviors.

Overall, our findings indicated a very low prevalence of individuals with recommended oral hygiene behaviors in Iran. We also found the presence of a relatively high degree of inequality in oral hygiene behaviors favoring individuals with higher SES. A possible explanation for the low prevalence of two studied hygiene behaviors may be due to the use of other oral hygiene aids in Iran including Miswak (salvadora persica), mouthwash etc. Unfortunately, there is no prevalence study on the use of Miswak in Iran at the national level and it would be interesting to examine the prevalence of Miswak use at a national level study.

A low prevalence of preventive oral hygiene behaviors indicated unsatisfactory adherence to oral hygiene behaviors in middle-aged and older adults in Iran. Previous studies [9, 30] also highlighted poor dental hygiene behaviors in Iran. We found that only 3.4% of middle-aged and elderly adults included in the study followed the two recommended hygiene behaviors. A study conducted in the general population in 2011 with 12,105 individuals in Iran reported a prevalence of 5.7% for both oral hygiene behaviors [9]. Our study showed a higher prevalence of brushing at least twice a day (64.6%) compared to that of the previous study in Iran (20.1%). This may indicate an improvement in adopting adequate tooth brushing behavior as a result of health education campaigns in Iran. Another explanation could be due to age differences between our study and the previously mentioned study in Iran (a mean age of 49.3 in our study versus a mean age of 37.8 in the previous mentioned one). This seems to be consistent with a study by Maes et al. which found that the prevalence of tooth brushing increased with increasing age, demonstrating an improvement in adopting the habit of brushing at least twice a day when young individuals were approaching adulthood [31]. We also found a prevalence of 15.9% for flossing behavior. This result is similar to that reported by the previous Iranian study [9] that found a prevalence of 16.8% for this hygiene behavior.

In addition to a low prevalence of oral hygiene behaviors, we found an unequal distribution of preventive dental self-care habits favoring individuals with higher SES in Iran. Recent studies also documented a significant increase in the differences in the oral health status of high and low SES individuals [19]. Part of oral health inequalities could be explained by this that high-SES are more likely to engage in healthy behaviors than individuals in low SES groups [32–34]. Previous studies conducted on socioeconomic inequality in the field of oral health in different countries generally indicated the presence of inequality in oral health status and behaviors [33, 35–37]. For example, consistent with our findings, a study conducted in Iran showed a pro-rich inequality in oral hygiene behaviors among children and adolescents [19]. Some studies have also indicated socioeconomic inequality in using dental care services and oral hygiene products such as toothbrushes and mouthwashes [33, 36, 38, 39]. A study in the UK also found a considerable socioeconomic inequality in oral health status favoring the better-offs [37]. Even though some previous research mentioned in this study were different in their oral health indicators as the outcome variables, the results of our study corroborate a pro-rich inequality in oral health indicators in general. Surprisingly, there was a pro-rich inequality for the dental flossing while a pro-poor gradient for tooth brushing was observed. A possible explanation for this is that more educated individuals and those with higher SES are probably more aware of oral health practices. In addition to using toothbrushes, they may also use other types of oral hygiene products compared to less educated and disadvantaged ones. On the other hand, less educated and lower-SES individuals probably are not aware of different oral hygiene products or they can only afford to pay for tooth brushing.

A primary and significant step towards reducing the observed socioeconomic inequality in oral hygiene behaviors is to estimate the contribution of determinants to such inequalities. Similar to a previous study [40], our decomposition analysis suggested SES and education level as the two main factors contributing to the observed inequality in oral hygiene behaviors. Apart from these two factors, the province of residence and age group made positive contributions to the socioeconomic-related inequality in oral hygiene behaviors. These results imply that the socioeconomic-related inequality in oral hygiene behaviors would have been reduced if these determinants had no impact on oral hygiene behaviors or were equally distributed across the SES groups. Socioeconomic status contributed to the concentration of oral hygiene behaviors among high SES individuals because, for example, higher SES allows individuals to pay for dental hygiene products or services, whereas the individuals of lower SES groups may not comply with the recommended oral hygiene behaviors due to their inability to pay for dental hygiene products or services [40]. Higher education level was another major contributor to pro-rich inequality in oral hygiene behaviors because highly-educated individuals are generally wealthier than less-educated ones and they are well-informed about the importance of tooth brushing and the use of dental floss for oral health gains [33] which, in turn, resulted in more adherence to preventive oral hygiene behaviors [41]. Interestingly, in a study by Chung et al., low-income individuals with a higher level of education reported better oral health behaviors including tooth-brushing and dental visits than high-income individuals with a lower level of education [41]. Particularly, health literacy has been shown to be associated with engaging in oral health-promoting behaviors and also oral health status [42, 43]. For example, Ueno et al. showed a significant association between oral health literacy and oral hygiene status and oral health behaviors [43]. It has been argued that a possible way to reducing oral health inequalities is improving the oral health literacy of all socio-demographic groups [44].

Our findings suggested that policy interventions for reducing inequalities in preventive oral hygiene behaviors should focus more on low-SES and less-educated adults in Iran. For example, providing special services to individuals of low-SES groups and presenting educational programs for improving oral health literacy especially in people with lower levels of education may mitigate socioeconomic inequality in the oral hygiene behaviors in Iran. Another possible explanation for socioeconomic inequalities in oral hygiene behaviors is psychosocial factors, e.g. psychological distress, work-related stress, social capital, and sense of coherence that have not been included in our study. These factors may play an additional role in explaining oral hygiene inequality and could be considered in future studies [12, 45–47].

A key strength of the study was that we used a large national sample to examine socioeconomic inequality in oral hygiene behaviors in cohort centers located in 14 different provinces in Iran. The large sample size gave us the opportunity to assess the regional variations in socioeconomic-related inequalities in oral hygiene behaviors in Iran. The novelty in the selection of middle-aged and older adults was another strength of the study. Nonetheless, our study has some limitations. First, although we used all the participants of the PERSIAN Cohort study in our analysis, the generalizability of our findings is limited because the PERSIAN Cohort study collects information from 14 out of 31 provinces in Iran. The results, therefore, need to be interpreted with caution. Second, due to the cross-sectional design of the study, we cannot establish the causality between determinants and oral hygiene behaviors in the decomposition analysis. Third, we used self-reported data on healthy oral hygiene behaviors may be affected by social desirability bias. Lastly, we had no access to data on diet habit at the time of the analysis. In addition, the information about dental visit has not been included in the questionnaire of the PERSIAN cohort.

## Conclusion

We found a low prevalence of oral hygiene behaviors in Iranian middle-aged and elderly adults. There was also pro-rich inequality in preventive oral hygiene behaviors among middle-aged and elderly adults in Iran. Socioeconomic status and level of education were the main factors contributed to the observed inequality, indicating that considering these factors could be useful in formulating public health policies to promote oral hygiene behaviors. The findings also suggested an urgent need for targeted policy interventions to encourage and improve oral hygiene behaviors among the poor and less-educated middle-aged and elderly adults in Iran.

## Data Availability

The datasets used and/or analysed during the current study are available from the corresponding author on reasonable request.

## Appendix

**Table 4:** The characteristics of cohort sites in Iran

Row	Province	Population	Cohort site	Population	Cohort population	Main Ethnicities
1	Ardabil	1,270,420	Ardabil	529,374	8192	Turk
2	Chaharmahal and Bakhtiari	947,763	Sharekord	93,104	6664	Lur
3	East Azerbaijan	3,909,652	Khameneh	3056	14,978	Turk, Azari
4	Fars	4,851,274	Kavar	31,711	2244	Fars (Persian), Turk
			Kharameh	18,477	10,662	Fars (Persian), Arab
			Fasa	110,825	10,113	Fars (Persian), Arab and Turk
5	Guilan	2,530,696	Some’e Sara	58,658	10,511	Gilaki
6	Hormozgan	1,776,415	Bandare Kong	19,213	3570	Arab
7	Kerman	3.164,718	Rafsanjan	161,909	9982	Fars (Persian)
8	Kermanshah	1,952,434	Ravansar	47,657	10,077	Kurd
9	Khouzestan	4,710,506	Hoveizeh	19,481	9156	Arab
10	Mazandaran	3,283,582	Sari	309,820	10,253	Tabari
11	Razavi Khorasan	6,434,501	Mashhad	3,001,184	2189	Fars (Persian)
			Sabzevar	243,700	784	Fars (Persian)
12	Sistan and Balouchestan	2,775,014	Zahedan	587,730	8318	Balouch
13	West Azerbaijan	3,265,219	Ghoushchi	2787	3662	Turk, Azari
14	Yazd	1,138,533	Shahedieh, Yazd	18,309	9901	Fars (Persian)

References: 1- Persian cohort sites, available from: http://persiancohort.com/cohortsites/, access: April 21, 2019. 2- Iran statistics center, available from: https://www.amar.org.ir, access: April 21, 2019

## Abbreviations

ADA: American Dental Association
C: Concentration index
PCA: Principal component analysis
PERSIAN: Prospective Epidemiological Research Studies in IrAN
SES: Socioeconomic status
C_n_: Normalized concentration index
